# Highlight: Genomic Insights into the Past and Future of the Black Rhinoceros

**DOI:** 10.1093/molbev/msad197

**Published:** 2023-09-14

**Authors:** Casey McGrath

The iconic African black rhinoceros (*Diceros bicornis*) faces an uncertain future after an intense poaching caused a 98% decline in wild populations from 1960 to 1995. Although numbers are currently increasing, the animal remains critically endangered. The historical range of the black rhinoceros covered vast swaths of sub-Saharan Africa, but today's remaining individuals inhabit just a handful of protected areas. The survival of the black rhinoceros within the fragmented remains of its natural habitat relies on dedicated conservation efforts. A new study published in *Molecular Biology and Evolution*, “Historic Sampling of a Vanishing Beast: Population Structure and Diversity in the Black Rhinoceros” (Sánchez-Barreiro et al. 2023), reshapes our understanding of the evolutionary and natural history of the black rhinoceros, opening a window into the species' genetic past while urging us to forge a path toward its conservation.

The study characterizes the population structure and genomic diversity of the black rhinoceros, both before and after its range-wide collapse in the last century, providing a model for how genetic diversity is shaped during population contractions. “The only way to really explore this is to use species with well documented, temporal collections that are also tied to good demographic records,” says Thomas Gilbert, one of the study's lead authors. “Sadly, species like the black rhinoceros are a perfect example, given their long-term appeal to big game hunters and poachers.” The motivation for the study, however, extended beyond mere scientific curiosity according to cofirst author Binia De Cahsan Westbury: “Studying the genetic history of the black rhinoceros through time provides crucial insights into its evolutionary trajectory and aids in developing effective conservation strategies for its remaining populations.”

With this goal, the authors sequenced the genomes of 63 museum specimens collected from 1775 to 1981, as well as 20 individuals from modern black rhinoceros populations, compiling the most comprehensive genetic dataset of the species to date and significantly advancing earlier research efforts. “Whole genome sequences have revealed much more conservation-relevant population structure in the black rhinoceros than expected from traditional markers,” notes the study's other lead author, Yoshan Moodley, emphasizing the transformative power of cutting-edge genomic techniques.

Analysis of the data revealed the presence of six major black rhinoceros populations historically as well as four subpopulations, offering more precise delineation of population borders than ever before. Notably, the results suggested that tectonic rifts in Africa during the Pleistocene had “driven the evolution of several hitherto unknown populations, many of which probably still exist within the present day Kenyan metapopulation,” highlights Moodley.

In addition to geographical barriers, the evolutionary history of the black rhinoceros was shaped by secondary contact when these barriers to gene flow were temporarily removed. “The interplay of these events has resulted in a significant pattern of isolation by distance across the sub-Saharan territory of the species,” says De Cahsan Westbury, referring to a trend in which populations that are farther apart geographically also show greater genetic differences from each other.

The researchers further evaluated levels of inbreeding among historical and modern populations of the black rhinoceros, an essential consideration for species that have suffered severe population bottlenecks. “Modern samples underscore the profound impact of population contractions and subsequent genetic drift,” notes De Cahsan Westbury, “with southern African individuals experiencing the most severe effects and the highest inbreeding among all populations.” Some populations showed evidence of inbreeding that predated the colonial period, which highlights the long-standing impact of human activity on this species according to the study's authors.

Altogether, the study offers a resounding call to action to improve the conservation and management of the black rhinoceros. “For too long, wildlife conservation authorities have struggled to incorporate and implement urgent genetic recommendations, to the detriment of the biodiversity concerned,” notes Moodley. “It is absolutely crucial that the new populations identified in East Africa be given the highest conservation priority,” he emphasizes, echoing the study's urgent plea for comprehensive genetic testing of black rhinoceroses in Kenya and Tanzania. In addition, the distinct evolutionary groups identified in the study, such as the Ruvuma, Maasai Mara-Serengeti, and possibly Chyulu National Park subpopulations, should be the focus of separate management to maintain their unique genetic lineages.

The study pays homage to the late Professor Mike Bruford of Cardiff University, a prominent figure in conservation genetics and a coauthor of the project. His death “was a tragedy for not only his family but conservation biology in general,” note the authors. Bruford's legacy continues to influence the field, an enduring testament to the pursuit of knowledge and the protection of Earth's genetic heritage.
